# Cerebrospinal Fluid YKL-40 and Neurogranin in Familial Alzheimer’s Disease: A Pilot Study

**DOI:** 10.3233/JAD-191261

**Published:** 2020-08-04

**Authors:** Steinunn Thordardottir, Ove Almkvist, Charlotte Johansson, Henrik Zetterberg, Kaj Blennow, Caroline Graff

**Affiliations:** a Department of NVS, Karolinska Institutet, Division of Neurogeriatrics, Center for Alzheimer Disease Research, Solna, Sweden; b Theme Aging, Karolinska University Hospital Huddinge, Unit for Hereditary Dementias, Solna, Sweden; c Department of NVS, Karolinska Institutet, Center for Alzheimer Research, Division of Clinical Geriatrics, Huddinge, Sweden; d Department of Psychiatry and Neurochemistry, Institute of Neuroscience and Physiology, The Sahlgrenska Academy at the University of Gothenburg, Mölndal, Sweden; e UCL Insitute of Neurology, Queen Square, London, UK; f UK Dementia Research Institute at UCL, London, UK; g Clinical Neurochemistry Laboratory, Sahlgrenska University Hospital, Mölndal, Sweden

**Keywords:** Alzheimer’s disease, biomarkers, cerebrospinal fluid, chitinases, genetics, mutation, neurogranin

## Abstract

**Background:**

YKL-40 and neurogranin are promising additional cerebrospinal fluid (CSF) biomarkers for Alzheimer’s disease (AD) which reflect different underlying disease mechanisms.

**Objective:**

To compare the levels of CSF YKL-40 and neurogranin between asymptomatic carriers of familial AD (FAD) mutations (MC) and non-carriers (NC) from the same families. Another objective was to assess changes in YKL-40 and neurogranin, from the presymptomatic to clinical phase of FAD.

**Methods:**

YKL-40 and neurogranin, as well as A*β*_42_, total tau-protein, and phospho-tau, were measured in the CSF of 14 individuals carrying one of three FAD mutations, *APPswe* (p.KM670/671NL), *APParc* (p.E693G), and *PSEN1* (p.H163Y), as well as in 17 NC from the same families. Five of the MC developed mild cognitive impairment (MCI) during follow-up.

**Results:**

In this pilot study, there was no difference in either CSF YKL-40 or neurogranin when comparing the presymptomatic MC to the NC. YKL-40 correlated positively with expected years to symptom onset and to age in both the MC and the NC, while neurogranin had no correlation to either variable in either of the groups. A subgroup of the participants underwent more than one CSF sampling in which half of the MC developed MCI during follow-up. The longitudinal data showed an increase in YKL-40 levels in the MC as the expected symptom onset approached. Neurogranin remained stable over time in both the MC and the NC.

**Conclusion:**

These findings support a positive correlation between progression from presymptomatic to symptomatic AD and levels of CSF YKL-40, but not neurogranin.

## INTRODUCTION

Alzheimer’s disease (AD) is the most common cause of dementia, with a prevalence of 4.4% in people over the age of 65 [1]. AD is characterized by protein aggregates in the form of plaques and cerebral amyloid angiopathy (amyloid-*β* peptide), neurofibrillary tangles, and neuropil threads (hyperphosphorylated tau protein), together with degeneration of neurons and loss of synapses [2]. The disease has a long preclinical phase, during which the patient is free of cognitive symptoms but exhibitsa series of biomarker changes related tounderlying AD pathology [3]. Our understanding today suggests that the first stage is defined by amyloidosis, detected either by low A*β*_42_ levels in the cerebrospinal fluid (CSF) or by high retention of an amyloid tracer evaluated by positron emission tomography (PET). During the second stage of preclinical AD signs of neurodegeneration appear in the form of high levels of CSF total tau-protein (t-tau) and phosphorylated tau-protein (p-tau). Finally, subtle signs of cognitive impairment appear, albeit not sufficient to meet the criteria for mild cognitive impairment (MCI) [4, 5].

The preclinical phase of AD is of high interest as it is believed to be the stage of the disease where therapeutic interventions are most likely to succeed. There still are many uncharted aspects of preclinical AD and the role and temporality of different events in its pathological cascade remain to be determined. Additional biomarkers representing immune responses, neurodegeneration, amyloid-*β* protein precursor (A*β*PP) metabolism and blood-brain-barrier function are all important to consider when characterizing this early stage of the disease [6].

Here, we studied asymptomatic carriers of autosomal dominant mutations leading to familial AD (FAD) and assessed the levels of two additional biomarkers, neurogranin and YKL-40, representing synaptic degeneration and glial activation respectively[6]. Both are of interest as potential biomarkers of early AD pathology and of possible treatment response. Asymptomatic carriers of FAD mutations are reliable models for the preclinical stage of AD, as they will develop symptoms of AD in the future with certainty, as FAD mutations are close to 100% penetrant [7, 8].

Neurogranin is a calmodulin-binding protein, expressed mainly in the dendritic spines of neurons in the association cortex of the brain [9, 10]. It is a postsynaptic protein, involved in synaptic plasticity [11, 12]. Loss of synapses is known to be a part of the pathological cascade of AD [13, 14], making a synaptic marker such as neurogranin a potentially important biomarker for disease pathogenesis. CSF neurogranin has been shown to be increased in patients with AD compared to control subjects in several studies [15–19] and has also been shown to be higher in persons with MCI who progress to AD dementia compared to persons with stable MCI [17, 20]. Further, one longitudinal study showed that high CSF levels of neurogranin predicted future cognitive decline in cognitively unimpaired elderly [21].

YKL-40, or chitinase-3-like protein, is aglycoprotein expressed by several cell types, including macrophages and vascular smooth muscle cells [22–24]. The functions of YKL-40 are still being elucidated but include regulation of inflammatory responses [25], promotion of cell proliferation and migration [26] and enhancement of tumor growth, angiogenesis, and macrophage infiltration [27]. In serum, it has been shown to be a promising diagnostic and prognostic biomarker for several clinical conditions such as cardiovascular disease, diabetes, and different types of cancer [28]. In AD, YKL-40 cell expression has been somewhat varied between studies and linked to both macrophages/microglia, astrocytes, and even to neurons [29–31]. A recent study on YKL-40 expression in human brain tissue identified a subset of astrocytes as the source of YKL-40 in AD and in tauopathies such as frontotemporal dementia [32]. High levels of YKL-40 in the CSF have been repeatedly shown to differentiate between AD patients and controls [29, 33–38], even if the degree of increase in AD is lower than that found for, e.g., CSF tau or neurofilament light protein [6]. Several studies have also shown higher YKL-40 levels in subjects with MCI-AD than in subjects with stable MCI [33, 36, 37].

The objective of this pilot study was to compare the levels of CSF YKL-40 and neurogranin between symptom free carriers of FAD mutations (MC) and non-carriers (NC) from the same families. A secondary objective was to assess the temporality of YKL-40 and neurogranin changes, from the presymptomatic to clinical phase of FAD, through longitudinal CSF sampling of both MC and NC. The limited sample size will of course also limit the generalizability of the results.

## MATERIALS AND METHODS

### Subjects at risk for FAD

The participants in this study are a part of a large longitudinal study on FAD that has been ongoing at Karolinska Institutet in Stockholm, Sweden, since 1993. They are members of three Swedish families, each carrying a different autosomal dominant mutation leading to FAD, the *APPswe* (p.KM670/671NL) mutation, the *APParc* (p.E693G) mutation and the *PSEN1* (p.H163Y) mutation. The FAD study is a prospective study of the natural course of FAD, where participants undergo repeated clinical evaluations, neuropsychological testing, MRI, electroencephalography, and biochemical assessments, including collection of CSF, blood, and fibroblasts [39–43]. Neuroimaging with PET using the radiotracers ^18^F-fluorodeoxyglucose, ^11^C-Pittsburg compound-B, and^11^C-deuterium-L-deprenyl is also included in the FAD study [44–47]. In this study the diagnosis of MCI is based on the recommendations from the International Working Group on Mild Cognitive Impairment from 2004 [48] and the diagnosis of dementia due to AD is based on the NIA-AA criteria [49]. The clinical diagnoses of individuals included in the study before the emergence of these criteria were re-evaluated after the new criteria were published. The family members are offered participation in the study after having approached the Unit for Hereditary Dementias at the Cognitive Clinic of the Karolinska University Hospital on their own initiative, or after having been contacted by a relative already enrolled in the study.

Here we present data from the subsample of participants in the FAD study (*n* = 31) who gave informed written consent to, and underwent, lumbar puncture on at least one occasion. All of the participants were free of cognitive symptoms at baseline and had a 50% risk of carrying one of the three mutations, *APPswe*, *APParc*, or *PSEN1* H163Y. The clinicians and researchers involved in the study were blind to the mutation status of the participants, as were the participants themselves, apart from those who had opted for presymptomatic genetic testing. One mutation carrier, aware of his mutation status, has been reported to be an outlier regarding CSF biomarkers in past studies that have included some of the same participants and therefore was excluded from the current study [39, 40].

All study procedures are in agreement with the Helsinki declaration and approved by the Regional Ethical Review Board in Stockholm, Sweden.

### CSF collection and analysis

The CSF samples were obtained in the time period between 1993 and 2015. CSF was collected into polypropylene tubes through lumbar puncture in the L3/L4 or L4/L5 interspace. The participants received premedication with 1 g paracetamol and 5 mg diazepam prior to the procedure. Immediately after collection, the CSF was centrifuged at 3000×g at +4°C for 10 min. The supernatant was pipetted off, aliquoted into polypropylene cryotubes and stored at –80°C.

All of the biomarkers included in the current study were measured at the same time, using the same batch of reagents, at the Clinical Neurochemistry Laboratory at the Sahlgrenska University Hospital, Mölndal, Sweden by board certified laboratory assistants, blind to clinical data.

CSF neurogranin was measured using a sandwich enzyme-linked immunosorbent assay (ELISA), developed in-house at the Sahlgrenska Clinical Neurochemistry Laboratory as described previously in detail [50]. CSF YKL-40 was measured using an YKL-40 ELISA kit, available from R & D Systems, Minneapolis, MN, USA. CSF A*β*_42_ was analyzed by the electro chemiluminescence technology (Meso Scale Discovery, Gaithersburg, Maryland, USA), using the MS6000 Human Abeta 3-Plex Ultra-Sensitive Kit [51]. CSF t-tau was determined using a sandwich ELISA (Innotesth TAU-Ag, Fujirebio Europe, Gent, Belgium) specifically constructed to measure all tau isoforms irrespectively of phosphorylation status, as previously described [52], while p-tau (tau phosphorylated at threonine 181) was measured using the Innotest® phospho-tau 181P ELISA (Fujirebio Europe, Ghent, Belgium) [53].

### Genetic analysis

#### Apolipoprotein E

The *APOE* genotyping was performed for SNPs rs7412 and rs429358 using TaqMan®, SNP Genotyping Assays (ABI, Foster City, CA, USA) according to manufacturer’s protocol. The amplified products were run on the 7500 fast Real-Time PCR Systems (ABI, Foster City, CA, USA).

#### Mutation analyses in APP and PSEN1

Exons 16 and 17 in *APP* were sequenced to screen for the KM670/671NL [54] and the E693G mutations [55]. To confirm the H163Y mutation in *PSEN1* exon 6 was sequenced [56]. DNA was amplified using AmpliTaq Gold® 360 PCR Master Mix (Applied Biosystems, Foster City, CA, USA). Primer sequences and PCR conditions are available upon request. Big Dye® terminator v3.1 Cycle sequencing Kit (Applied Biosystems, Austin, TX, USA) was used for Sanger sequencing. The exons in *APP* and *PSEN1* were sequenced in both directions and analyzed on an ABI3500 Genetic Analyzer (Applied Biosystems, Foster City, CA, USA).

### Statistical analysis

The study group was divided into an MC group and an NC group. The two groups were compared with regards to age, expected years to onset (see below), gender distribution, number of carriers of the *APOEɛ*4 and *APOEɛ*2 genotypes, and CSF levels of YKL-40, neurogranin, t-tau, p-tau, and A*β*_42_. The D’Agostino & Pearson normality test was used to assess the distribution of variables. For normally distributed variables we used unpaired *t*-test to compare groups, while the Mann-Whitney U-test was used for comparing variables that were not normally distributed. Fisher exact test was used for categorical variables.

Correlations were made between YKL-40 and t-tau, p-tau, and A*β*_42_, as well as between neurogranin and t-tau, p-tau, and A*β*_42_. Correlations were also made betweenYKL-40, neurogranin, and expected years to onset. Here, the Pearson correlation coefficient was used for normally distributed data, otherwise the Spearman correlation coefficient was used.

To correct for multiple comparisons, we used the Benjamini and Hochberg FDR correction method with Q set to 5% [57].

Longitudinal CSF samples were available from a subgroup of the study participants. In the longitudinal analysis a two-way (MC versus NC) ANOVA was used to compare the mean of annual change in YKL-40 and neurogranin (last –first YKL-40/neurogranin value divided by number of years between first and last value) between MC and NC.

### Expected years to symptom onset

Age at symptom onset is defined as the age at which an individual experiences the first documented clinically relevant cognitive symptoms. The mean age at symptom onset and standard deviation for affected family members in each family included here is 54±4 years for *APPswe* (based on 19 affected cases), 56±4 years for *APParc* (based on 12 affected cases), and 51±7 years for *PSEN1* H163Y (based on 11 affected cases). If an individual has not reached the age at symptom onset in his or her family, the value for expected years to symptom onset will be negative. For example, the expected years to symptom onset for a 46-year-old from the *PSEN1* H163Y family will be 46 years –51 years = –5 years to expected symptom onset.

Expected years to symptom onset suggests each mutation carrier’s position on the continuum of the pathological cascade of AD with the onset of cognitive symptoms as a point of reference. Even though the non-carriers serve as healthy controls and are not expected to develop cognitive symptoms around the mean onset age in their families, calculating expected years to symptom onset is still motivated in the NC group. Calculating expected years to symptom onset in the NC enables us to assess when, in relation to the age of expected family specific symptom onset, the CSF biomarker levels of the MC start to deviate from the NC.

## RESULTS

### Demographics of the study population

A total of 31 individuals participated in the study, 14 were mutation carriers and 17 were non-carriers. YKL-40 was assessed in the CSF samples from all 31, but due to lack of volume of a few of the samples t-tau levels were available from 29 of the participants, A*β*_42_ levels from 27 participants and p-tau and neurogranin levels from 25 participants.

When the whole group of 31 subjects was divided into an MC group and an NC group, the groups did not differ significantly with regards to age, gender, and expected years to symptom onset. Also, the number of carriers of the*APOEɛ*4 and *APOEɛ*2 genotypes was not significantly different between groups. In the MC group, there were 4 carriers of the *APPswe* mutation, 4 carriers of the *APParc* mutation, and 6 carriers of the *PSEN1* H163Y mutation. The demographic characteristics of the carriers of each specific mutation will not be revealed in more detail for the sake of anonymity. The demographic data of the whole study population, as well as levels of the traditional AD CSF-biomarkers A*β*_42_, t-tau, and p-tau, are presented in Table 1.

**Table 1:** Demographic data and CSF-biomarker levels of the included subjects at risk for FAD. Demographic characteristics of the mutation carrier group and the non-carrier group at the time of baseline CSF sampling. Age, expected years to symptom onset, and levels of A*β*42, YKL-40, and neurogranin are presented as mean values ± standard deviation. *Levels of t-tau and p-tau are presented as median with range, due to lack of normal distribution of the data. One of the mutation carriers is an APOE e2/e4 heterozygote and is included in the table as both an *ɛ*2 and an *ɛ*4 carrier. N, number; NS, not significant

	Mutation carriers	Non-carriers	*p*
	(*n* = 14)	(*n* = 17)
Age	43±10	46±12	NS
Expected years to symptom onset	–12±10	–9±12	NS
Gender (Male/Female)	11/3	10/7	NS
*APOEɛ*4 carriers (n)	8	7	NS
*APOEɛ*2 carriers (n)	1	3	NS
A*β*_42_ (pg/mL)	221±138	626±277	*p* < 0.0001
t-tau* (pg/mL)	472 (169–937)	241 (122–537)	*p* = 0.01
p-tau* (pg/mL)	58 (28–104)	36 (19–76)	*p* = 0.06
YKL-40 (ng/mL)	98±33	91±38	NS
Neurogranin (pg/mL)	252±90	215±84	NS

Age, expected years to symptom onset, distribution of gender, *APOEɛ*4 carriers, and *APOEɛ*2 carriers were also compared between the groups of MC and NC having samples that were analyzed for neurogranin, t-tau, p-tau, and A*β*_42_. There was no significant difference between any of these variables in any of the MC versus NC groups that were analyzed for the aforementioned biomarkers. Of the 25 study participants who had enough sample volume to analyze neurogranin, 11 were MC and 14 were NC. The mean age of the MC group with neurogranin was 45±8 years, the same mean age as in the NC group with neurogranin (45±13 years). The mean number of expected years to symptom onset in the neurogranin MC group was –10±8 years and –10±13 years in the neurogranin NC group.

Finally, due to two previously reported cases of decreased penetrance of the *PSEN1* H163Y mutation [58] (one of which was the outlier mentioned in the introduction), we report the CSF biomarker and clinical status of the six *PSEN1* H163Y mutation carriers in the current study; Four of the *PSEN1* H163Y mutation carriers had more than one CSF samples and all four had low levels of CSF A*β*_42_ and high levels of CSF t-tau and p-tau, suggesting AD pathological change and penetrance of the mutation. Two of the *PSEN1* H163Y mutation carriers had only one CSF sample each, one of which had low levels of CSF A*β*_42_ (suggesting penetrance), while the other had normal levels of all of the three core AD CSF biomarkers. This last individual has since the time of CSF sampling developed cognitive symptoms suggestive of clinical AD. Therefore, there is nothing that suggests that the *PSEN1* H163Y mutation carriers included in the current study have reduced penetrance of the mutation.

### Baseline levels of YKL-40 and neurogranin in the CSF

The mean level of YKL-40 the mutation carrier group was 98 ng/mL, with a standard deviation (s.d.) of 33. The mean YKL-40 level in the non-carriers was 91 ng/mL, with an s.d. of±38. There was no significant difference between the levels of YKL-40 in the two groups. The same applied when levels of neurogranin were compared between the MC and NC. The difference between the groups was not significant, with a mean neurogranin level of 252±90 pg/mL in the MC and 215 pg/mL±84 in the NC (see Table 1). Finally, the levels of YKL-40 and neurogranin were compared between the carriers of the three different FAD mutations included in the study. There were no significant differences in the levels of either biomarker when comparing the carriers of each specific mutation to the carriers of the other mutations (data not shown).

### Correlations between CSF YKL-40, CSF neurogranin, and CSF t-tau, p-tau, and A*β*_42_

**Fig. 1:**
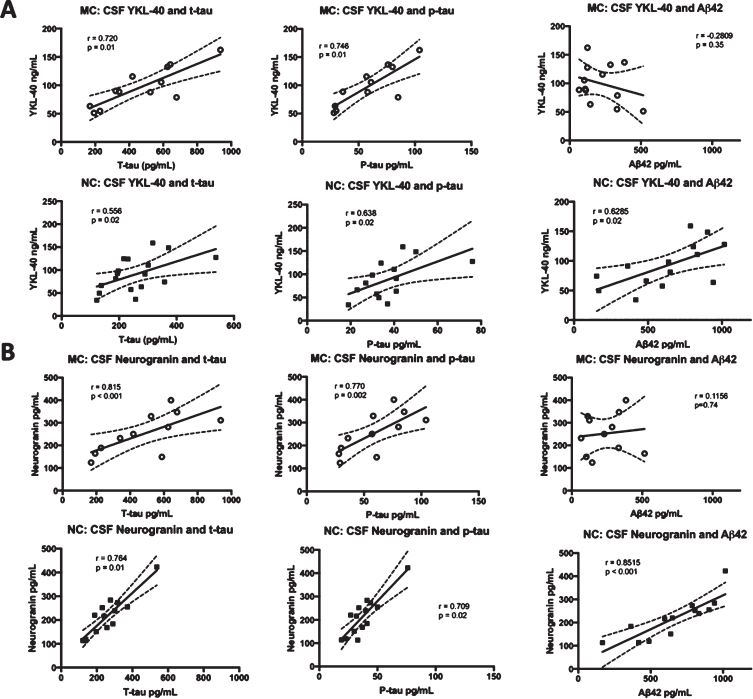
Correlations between the levels of CSF YKL-40 and neurogranin versus t-tau, p-tau, and A*β*_42_. Correlations between CSF YKL-40 (A) and neurogranin (B) and the clinically established CSF biomarkers t-tau, p-tau, and A*β*_42_ in FAD MC and NC. The solid lines indicate the linear-regression, the dotted lines represent the 95% confidence interval and the “r” represents the Pearson correlation coefficient when correlating A*β*_42_ to YKL-40 and neurogranin and the Spearman correlation coefficient when correlating t-tau and p-tau to YKL-40 and neurogranin. All the correlations were positive and significant except for the correlation between YKL-40 and A*β*_42_ in the MC group and between neurogranin and A*β*_42_, also in the MC group. The symbols represent individual values (filled symbols NC and white symbols MC) CSF, cerebrospinal fluid; FAD, familial Alzheimer’s disease; MC, mutation carriers; NC, non-carriers.

Correlations were made between CSF YKL-40 and neurogranin levels and the levels of the three CSF biomarkers of AD that are in clinical use, t-tau, p-tau, and A*β*_42_ [59]. CSF YKL-40 correlated positively with t-tau (*r* = 0.8335, *p* < 0.001) and p-tau (r = 0.8296, *p* = 0.002) in the MC. However, the correlation between YKL-40 and A*β*_42_ (r = –0.2809, *p* = 0.35) was not significant in the MC group. In the NC, YKL-40 correlated positively with all three biomarkers, t-tau (r = 0.5415, *p* = 0.02), p-tau (r = 0.5868, *p* = 0.03),and A*β*_42_ (r = 0.6285, *p* = 0.02). There were positive and significant correlations between neurogranin and t-tau and p-tau in both MC (r = 0.699, *p* = 0.02 and r = 0.727, *p* = 0.01, respectively) and NC (r = 0.902, *p* < 0.0001 and *r* = 0.866, *p* < 0.0001, respectively). Furthermore, there was a positive correlation between neurogranin and A*β*_42_ in the NC (r = 0.8515, *p* < 0.001) but not the MC (r = 0.1156, *p* = 0.74). See Fig. 1 for an overview of the correlations between CSF biomarkers.

### Correlations between expected years to symptom onset, age, and the CSF biomarkers YKL-40 and neurogranin

**Fig. 2:**
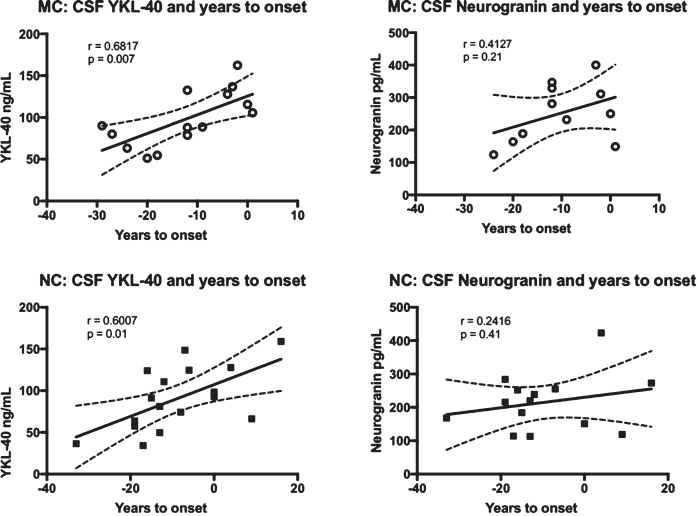
Correlations between the levels of YKL-40 and neurogranin versus expected years to symptom onset in FAD MC and NC. Correlations between CSF YKL-40, neurogranin and expected years to symptom onset in FAD MC and NC. The solid lines indicate the linear regression and the dotted lines represent the 95% confidence interval. The “r” represents the Pearson correlation coefficient. The symbols represent individual values (filled symbols NC and white symbols MC).CSF, cerebrospinal fluid; FAD, familial Alzheimer’s disease; MC, mutation carriers; NC, non-carriers.

To see if YKL-40 and/or neurogranin correlated with the approach of symptoms, we correlated these markers with expected years to symptom onset. In the MC group, YKL-40 correlated positively with expected years to symptom onset (r = 0.6817, *p* = 0.007), while the correlation between neurogranin and expected years to symptom onset was not significant. The same results were found for the NC group, with YKL-40 again correlating positively with expected years to symptom onset (r = 0.6007, *p* = 0.01). See Fig. 2 for a summary of the correlations between the CSF biomarkers and expected years to symptom onset.

Years to expected symptom onset is a good surrogate for age, but to better establish the relationship between YKL-40, neurogranin, and advancing age, we correlated age at baseline CSF sampling directly to these two markers. Age turned out to be positively correlated with YKL-40 levels in both the MC (r = 0.6811, *p* = 0.007) and the NC (r = 0.6523, *p* = 0.005). Neurogranin neither had a correlation to age in the MC nor the NC.

The correlations between the different biomarkers (as presented in Fig. 1), between the biomarkers and expected years to symptom onset (as presented in Fig. 2), and between the biomarkers and ageat baseline were corrected for multiple comparisons using FDR correction. All of the significant correlations remained significant after the correction for multiple comparisons.

### Longitudinal data on CSF YKL-40 and neurogranin levels

Nine of the fourteen MC underwent repeated CSF sampling, with the first follow-up sample taken 5 years from baseline on average. Six participants were followed-up once, while three underwent more than one follow-up CSF sampling. Four of the participants in the MC group had developed MCI at first follow-up and one more developed MCI at second follow-up, with the remaining four continuing to be symptom free.

In the NC group, five of the seventeen participants returned for follow up CSF sampling, 6 years on average from baseline. Four of the NC had one follow-up sampling while one NC had two. All of the NC were symptom free at all follow-up visits.

There was no significant difference in age, expected years to symptom onset, gender distribution, or number of *APOEɛ*4 or *APOEɛ*2 carriers between the nine MC and the five NC who had more than one CSF sample (data not shown).

Levels of YKL-40 were analyzed in all samples from the nine MC and five NC who had longitudinal samples. The sample volume from one of the MC was not sufficient to analyze neurogranin as well, resulting in longitudinal neurogranin levels being available from eight MC and five NC. The MC and the NC groups analyzed for longitudinal neurogranin levels did not differ significantly with regards to age, expected years to symptom onset, gender, *APOEɛ*4- or *APOEɛ*2-status (data not shown).

**Fig. 3:**
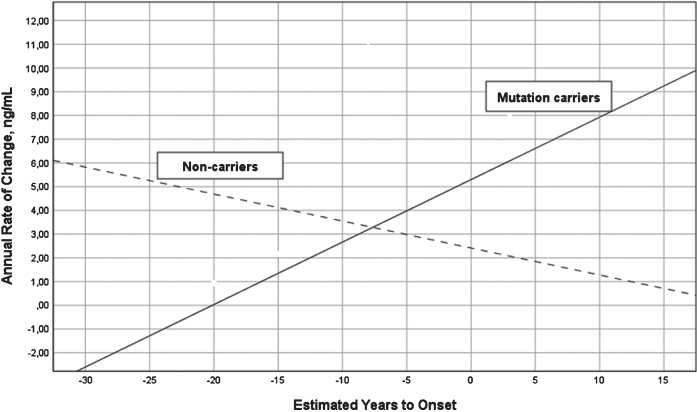
Annual change in YKL-40 levels versus expected years to onset in FAD MC and NC. Annual change in the levels of CSF YKL-40 in FAD MC (solid line) and NC (dashed line) in reference to expected years to symptom onset. The mean annual change in CSF YKL-40 differed significantly between the MC and NC (F = 13.18, *p* = 0.005). CSF, cerebrospinal fluid; FAD, familial Alzheimer’s disease; MC, mutation carriers; NC, non-carriers.

A two-way ANOVA with group (MC versus NC) and stage based on expected years to symptom onset (preclinical versus postclinical) showed that the interaction between group and stage was significant for annual change in YKL-40 (last –first YKL-40 value divided by number of years between first and last value) (F = 9.71, df = 1/10, *p* = 0.011, eta^2^ = 0.49), but not main effects of group or stage. The annual change increased in MC (r = 0.78, df = 10, *p* = 0.013) and decreased in NC (r = –0.64, df = 3, *p* > 0.1) as in relation to expected years to symptom onset, see Fig. 3. The annual change was best described by a linear regression model, other models were not significant. The same longitudinal analysis on neurogranin was not significant.

## DISCUSSION

This study showed no difference in the levels of CSF YKL-40 or neurogranin when comparing asymptomatic FAD MC to NC from the same families. The MC for which levels of YKL-40 were available had an average of 12 years left to the expected onset of first clinical symptoms, while the MC subgroup, having enough sample volume to analyze neurogranin as well, had an average of 10 years left to expected symptom onset. Regarding CSF YKL-40, these results are not in concordance with the results of a large study on CSF biomarkers in the Dominantly Inherited Alzheimer Network (DIAN), which showed an increase in CSF YKL-40 in FAD mutation carriers 15–19 years before the expected onset of clinical symptoms [60]. No presymptomatic change in CSF neurogranin was observed in the DIAN study, which is in agreement with the result obtained in the current study. There could be several reasons for this discrepancy regarding YKL-40, including that the current study did not involve carriers of the same FAD mutations as the DIAN study. Also, the DIAN study has a larger number of participants, thereby increasing its power to detect differences in the levels of CSF biomarkers.

Studies on CSF YKL-40 and neurogranin in sporadic AD published to date have not been in agreement regarding the temporality of changes in these biomarkers. One study found no change in CSF YKL-40 levels in individuals with reduced levels of A*β*_42_but no cognitive or psychiatric symptoms, indicating that they were in the preclinical stage of AD, while high YKL-40 levels were found in a patient group with abnormal AD biomarkers (A*β*_42_, t-tau, and p-tau) and subtle memory deficits, classified as prodromal AD [61]. Another study produced similar results, with an increase in CSF YKL-40 levels observed in the MCI and dementia stages of AD, but not in subjects with subjective cognitive impairment and pathological levels of CSF A*β*_42_ [62]. Janelidze et al. reported high levels of YKL-40 in the preclinical, MCI, and dementia stages of AD [63]. Finally, in a recent study by Bos et al., individuals with no cognitive symptoms and decreased levels of CSF A*β*_42_ had increased levels of both CSF YKL-40 and neurogranin. Here, high YKL-40 levels were observed only in the preclinical stage of sporadic AD, while neurogranin levels remained high in the MCI and dementia stages [64]. Also, high levels of neurogranin have been reported in cognitively healthy individuals who later experienced a decline in cognition, as well as in healthy older subjects (with a mean age of 83 years) who had low CSF A*β*_42_ levels, indicative of preclinical AD, compared to those who did not have low CSF A*β*_42_ levels [65]. A longitudinal increase in CSF neurogranin levels has also been linked to a decline in white matter health, observed through diffusion tensor imaging, in late middle-aged adults [66]. The explanation for the discrepancy between our results and the results of the neurogranin studies mentioned above is not clear, but the lack of statistical power due to the small sample size in our study could play a role. Also, this could possibly be related to our group of subjects being younger, with a lower burden of central nervous system (CNS) comorbidities, and possibly less advanced in the presymptomatic stage of AD than the subjects in the other studies. The finding that neurogranin levels did not increase as the expected age at symptom onset approached does make the latter explanation unlikely however. Also, the MC in this study had low levels of A*β*_42_ *and* increased t-tau, indicating that they were past the initial stages of presymptomatic AD.

In the current study, YKL-40 and neurogranin correlated positively with t-tau and p-tau in the MC, but there was no correlation between these two experimental markers and A*β*_42_in the MC group. A*β*_42_ has been shown to decrease early in preclinical AD and remain stable and low thereafter [39, 67–69], which probably explains the lack of correlation with A*β*_42_. Interestingly, there was a positive correlation between both neurogranin and YKL-40 and all of the three AD biomarkers, t-tau, p-tau, and A*β*_42_ in the NC. This comes as a surprise as t-tau and p-tau increase over time in AD but A*β*_42_ decreases and then stabilizes at a low level. One would therefore expect no correlation, or a negative one, between A*β*_42_ and YKL-40 and neurogranin if these two markers were reflecting AD pathology in the same way as A*β*_42_. As t-tau is considered to be less specific for AD than p-tau [59], the positive correlation between t-tau and YKL-40 and neurogranin in the NC could be explained by all three markers reflecting inflammation and/or neuronal damage related to processes other than AD, such as aging. Finding the same positive correlation with p-tau in the NC is more difficult to explain. One could argue that some of the NC might indeed be in the preclinical stage of AD, as it is a common disease in the general population and does not require the presence of a specific mutation. However, a negative (or no) correlation to A*β*_42_ in the NC would be expected if that were the case.

YKL-40 correlated positively with expected years to symptom onset in both the MC and the NC, while no such correlation was found for neurogranin. Based on these results it is not possible to link either biomarker to early AD progression and this even suggests that YKL-40 might be an unspecific marker of the ageing process. The longitudinal data on YKL-40 also showed an increase in YKL-40 levels in the MC as the expected age at symptom onset approached, but not in the NC. Therefore, there is a discrepancy between the cross-sectional data and the longitudinal data regarding the change in YKL-40 levels in relation to expected symptom onset in the NC. The most likely explanation for this is the small number of participants in the longitudinal part of the study which decreases its power. The cross-sectional data should also be interpreted with caution as cross-sectional data gives more limited information on changes over time than longitudinal data. With these limitations in mind the cross-sectional data suggests that YKL-40 might be a marker of a process related to normal aging which is exacerbated by a concomitant AD pathology. The same age dependent increase in CSF levels of YKL-40 has been observed previously in cognitively healthy middle-aged individuals, with a sharper increase in carriers of the *APOEɛ*4 allele than in non-carriers [67]. An increase in YKL-40 has been reported in AD dementia as well as in frontotemporal dementia and YKL-40 has also been associated with other inflammatory processes in the CNS, such as multiple sclerosis [70, 71]. YKL-40 expression has been shown to be markedly increased in astrocytes in the acute phases of cerebral infarction [71], further underpinning its possible role as an unspecific marker of CNS damage and aging. Interestingly, a recent study including data from the Alzheimer’s Disease Neuroimaging Initiative (ADNI) showed decreasing levels of both YKL-40 and neurogranin with the progression of symptomatic late onset sporadic AD [72]. These results indicate that the picture is more complex, with neuroinflammation and synaptic loss waxing and waning throughout the AD continuum.

Here, there were no signs of CSF neurogranin increasing over time in either the MC or the NC according to the longitudinal analysis. It is the rule, rather than the exception, that brains of elderly individuals exhibit multiple pathologies on autopsy, including loss of synapses [73]. Here we have a young group of subjects who we expect to be mostly free of comorbidities which might contribute to an increase in CSF neurogranin. From our results, it seems that presymptomatic AD alone is not enough to cause an increase in neurogranin, but adding other comorbidities might produce a synergistic effect explaining the neurogranin increase in AD seen in other studies.

A limitation to this study is the relatively small sample size, which reduces the power of the study to detect true differences between the MC and NC groups, and this should be taken into account when interpreting the data. However, despite this small sample size, significant differences were observed between the MC and NC in the levels of CSF A*β*_42_ and t-tau, which indicates that the current study has the power to detect differences between the groups regarding these traditional biomarkers. This might indicate that YKL-40 and neurogranin do not separate the two groups as well in this early presymptomatic stage as A*β*_42_ and t-tau.

Also, there was a predominance of males in both the MC and NC groups which could possibly confound the results. The longitudinal results should be interpreted with some caution due to the small number of longitudinal samples, differences in age at baseline sampling between individuals and differences in time interval until a follow-up sample was obtained. Despite these limitations, a longitudinal analysis of these biomarkers strengthens the current study. The need for more longitudinal studies on this subject is underlined by the somewhat contradictory results from the numerous studies on YKL-40 and neurogranin in sporadic AD presented above. These additional AD biomarkers need to be studied longitudinally in different populations, through different stages of the disease, as these markers seem to be presenting a more nuanced and complex sequence of events in AD than the clinically established biomarkers A*β*_42_, t-tau, and p-tau which show a robust and predictable signal in all stages of FAD and sporadic AD.

In conclusion, this study suggests that neither CSF YKL-40 nor neurogranin are very early presymptomatic biomarkers of AD. According to our results YKL-40 rises with increasing age in FAD MC and possibly also in NC, albeit not as steeply, starting before the symptom onset of AD. Charting this rise might serve as an alert to timely intervention, if and when available. There was no such signal for neurogranin, which did not separate the MC from the NC and was stable over time in both groups.
